# Vitrimers: permanent organic networks with glass-like fluidity

**DOI:** 10.1039/c5sc02223a

**Published:** 2015-10-08

**Authors:** Wim Denissen, Johan M. Winne, Filip E. Du Prez

**Affiliations:** a Department of Organic and Macromolecular Chemistry , Polymer Chemistry Research Group and Laboratory for Organic Synthesis , Ghent University , Krijgslaan 281 S4-bis , B-9000 , Ghent , Belgium . Email: johan.winne@ugent.be ; Email: filip.duprez@ugent.be

## Abstract

Most covalent adaptable networks give highly interesting properties for material processing such as reshaping, recycling and repairing. Classical thermally reversible chemical cross-links allow for a heat-triggered switch between materials that behave as insoluble cured resins, and liquid thermoplastic materials, through a fully reversible sol–gel transition. In 2011, a new class of materials, coined vitrimers, was introduced, which extended the realm of adaptable organic polymer networks. Such materials have the remarkable property that they can be thermally processed in a liquid state without losing network integrity. This feature renders the materials processable like vitreous glass, not requiring precise temperature control. In this mini-review, an overview of the state-of-the-art in the quickly emerging field of vitrimer materials is presented. With a main focus on the chemical origins of their unique thermal behavior, the existing chemical systems and their properties will be discussed. Furthermore, future prospects and challenges in this important research field are highlighted.

## Abstract 2

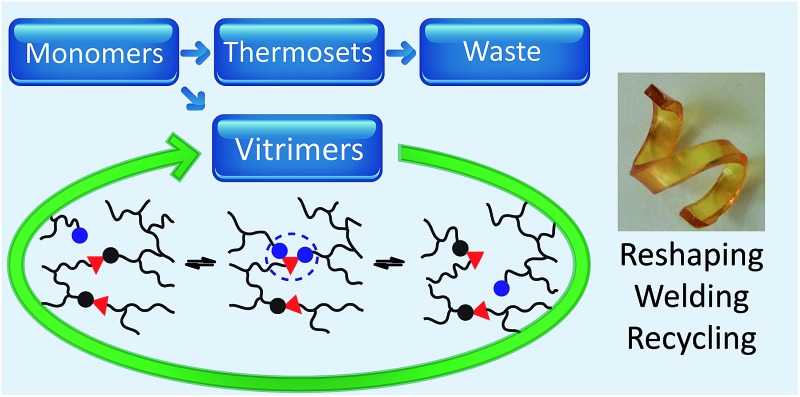
Vitrimers possess the unique property that they are malleable while being permanently cross-linked. This mini-review highlights the existing vitrimer systems in the period 2011–2015 with the main focus on their chemical origin.

## Introduction

Thermosets are the materials of choice for numerous applications because of their dimensional stability, mechanical properties and creep/chemical resistance. However, as a result of their permanent molecular architecture, thermosets cannot be reshaped, processed or recycled.

An attractive chemical strategy to introduce plasticity in cross-linked polymer networks is offered by the introduction of exchangeable chemical bonds, leading to dynamic cross-links. If chemical cross-links can be efficiently and reliably exchanged between different positions of the organic polymer chains, macroscopic flow can be achieved without risking structural damage or permanent loss of material properties. Polymer networks containing such exchangeable bonds are also known as covalent adaptable networks or CANs and have been recently reviewed by Bowman and Kloxin.^1–3^

**Fig. 1:**
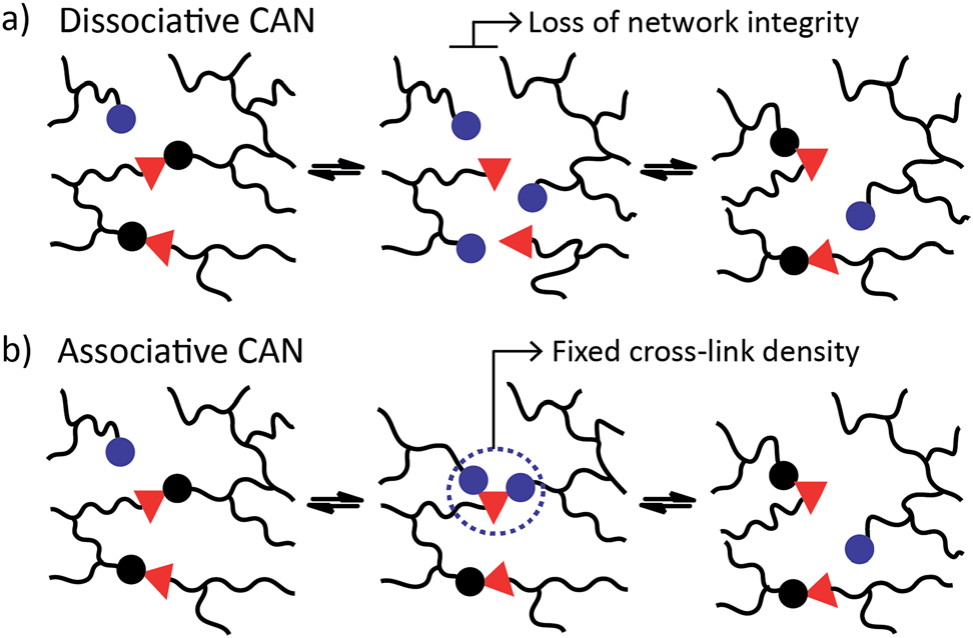
CANs are divided in two groups: (a) dissociative, and (b) associative, based on the exchange reactions that proceed respectively with or without a temporary net loss of cross-link density. Vitrimers belong to the group of associative CANs.

CANs may be further classified into two groups depending on their exchange mechanism. The first group of CANs makes use of a *dissociative* cross-link exchange mechanism. In this exchange, chemical bonds are first broken and then formed again at another place (Fig. 1a). The second group of CANs makes use of *associative* bond exchanges between polymer chains, in which the original cross-link is only broken when a new covalent bond to another position has been formed (Fig. 1b).

A well-explored chemical system for the design and synthesis of dissociative CANs is the reversible Diels–Alder reaction between furans and maleimides in organic polymer networks. Upon heating, this only mildly exothermic (5–10 kcal mol^–1^) Diels–Alder cross-linking reaction becomes reversible, leading to an increased rate of bond breaking/reforming and also to a net bond dissociation because the chemical equilibrium shifts relatively towards the endothermic side. Thus, such materials can achieve very fast topology rearrangements (stress relaxation and flow) because of a decrease in connectivity. This temporary loss of cross-links typically results in a sudden viscosity drop, as is normally observed for thermoplastic materials. Upon cooling, the cross-links are formed again, usually to the same extent as in the starting material, thus preserving or re-installing the desirable thermosetting properties such as stiffness and insolubility. In this way, these dynamic cross-links allow for thermal (re-)processing of polymeric networks.

The second group of CANs, polymer networks that rely on associative exchange mechanisms, do not depolymerise upon heating, but are characterized by a fixed cross-link density. Covalent bonds are only broken when new ones are formed, making these networks permanent as well as dynamic. The first reported associative CANs (2005) were based on photo-mediated free radical addition fragmentation chain transfer reactions by using moieties such as allyl sulphides.^4,5^ Later, a similar exchange mechanism was introduced in CANs by using alternative radical generators with trithiocarbonates.^6–8^ Despite showing interesting flow and stress-relaxation, the ultimate adaptability or dynamic lifetime of these systems is limited due to the radical nature of the involved reactions, which gives rise to unavoidable termination reactions.

In 2011, Leibler and co-workers extended the realm of associative CANs by adding a suitable transesterification catalyst to epoxy/acid or epoxy/anhydride polyester-based networks.^9^ This thermally triggered catalytic transesterification reaction resulted in permanent polyester/polyol networks that show a gradual viscosity decrease upon heating, a distinctive feature of vitreous silica,^10^ which had never been observed in organic polymer materials. Hence, the authors introduced the name vitrimers for those materials.

Since then, several variations and alternative chemistries that can induce this remarkable thermal behavior have been explored. Following a brief discussion of the unique properties of vitrimers from a polymer chemistry perspective, this mini-review will provide an overview of the existing chemical systems for vitrimers and vitrimer-like materials. We hope that this will spur further advances and opportunities for collaboration and innovation in this emerging field at the interface of material science, polymer science and organic reactivity. For this, the materials are discussed and categorised based on the chemical moieties and types of reactivity that are responsible for the dynamic exchange reactions, rather than by the more classical polymer classification based on the chemical nature of the bulk of the polymer material.

## Vitrimers – characteristics and chemical roots

**Fig. 2:**
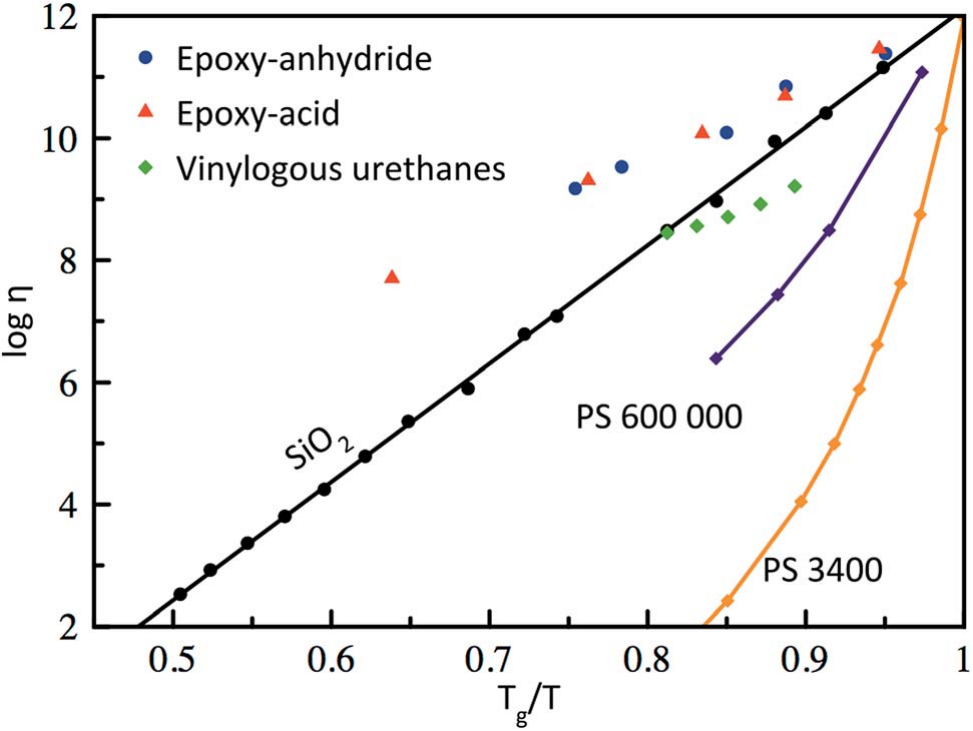
Angell fragility plot, showing the viscosity as a function of the inverse temperature, scaled with *T*_g_ (or *T*_v_ for vitrimers when *T*_v_ > *T*_g_). Thermoplastics such as polystyrene (PS)^16^ are characterized with a narrow glass transition temperature and thus a very fast decrease in viscosity near *T*_g_. In contrast, vitrimers (epoxy-anhydride based, epoxy-acid based^14^ and vinylogous urethanes^17^) show an Arrhenius-like dependence of the viscosity, which results in a gradual viscosity decrease similar to vitreous silica.^18^

Based on the pioneering work of Leibler *et al.*,^9,11–14^ this new class of materials can be defined by some criteria. First, vitrimers are made of covalently bound chains forming an organic network. This network is furthermore able to change its topology *via* exchange reactions that are associative in nature and thermally triggered, resulting in the thermal malleability of the network. At higher temperatures, the viscosity of vitrimers is essentially controlled by chemical exchange reactions, giving a thermal viscosity decrease that follows the Arrhenius law, as is observed in typical inorganic silica materials. This latter property distinguishes vitrimers from dissociative CANs and thermoplastic materials because these materials evolve from a solid to a liquid state in a much more abrupt way, following the Williams–Landel–Ferry model (WLF) for thermoplastic polymer melts (Fig. 2).

As vitrimers are permanent networks with a permanent connectivity at all temperatures (excluding degradation), these materials swell but do not dissolve in chemically inert solvents, even when heated. In contrast to classical polymer networks, swelling ratios can be expected to be higher since the elastic retractive forces, opposing the increase in entropy and heat of mixing associated with polymer swelling, can be relaxed due to topology rearrangements.

The viscoelastic behavior of vitrimers can be described using two transition temperatures. The first one is the usual glass-transition temperature, *T*_g_, between the glassy and rubbery state of polymer networks, correlated to the onset of long-range, coordinated molecular motion. The second transition temperature derives from the network cross-link exchange reactions. When the timescale of bond exchange reactions becomes shorter than the timescale of material deformation, the network can rearrange its topology, resulting in flow. Hence, a transition from viscoelastic solid to viscoelastic liquid occurs at a temperature denoted as the topology freezing transition temperature, *T*_v_ by Leibler *et al.* This transition is conventionally chosen at the point where a viscosity of 10^12^ Pa s is reached.^9,15^ *T*_v_ can also be observed experimentally by dilatometry, since a reorganising network has a higher expansion coefficient than a static network.

**Fig. 3:**
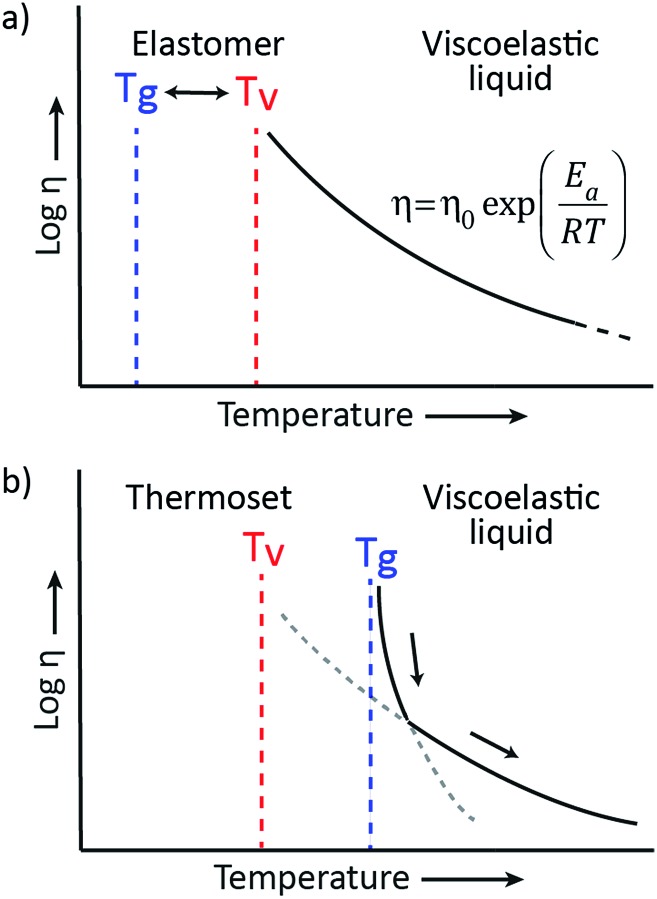
Representation of the viscoelastic behavior of vitrimers with (a) a glass transition, *T*_g_, lower than the topology freezing transition temperature, *T*_v_. Upon heating, the vitrimer evolves from a glassy solid (*T* < *T*_g_) to an elastomer (*T*_g_ < *T* < *T*_v_) to a viscoelastic liquid (*T* > *T*_v_) that follows the Arrhenius law. (b) A hypothetical *T*_v_ is situated well below *T*_g_. Upon heating, the vitrimer evolves from a glassy solid to a viscoelastic liquid with a viscosity that is first controlled through diffusion (WLF) and then by the exchange kinetics (Arrhenius).

The two transition temperatures, characteristic for vitrimer materials, and their relevance can best be clarified using two distinct examples. In the first example, the vitrimeric system has a *T*_g_ lower than *T*_v_ (Fig. 3a). Upon heating from a temperature below *T*_g_ to a temperature between *T*_g_ and *T*_v_, the glassy solid will first undergo a transition to the rubbery state and will behave as an elastomer since the exchange reaction is so slow that the network structure is essentially fixed. Only on further heating, the exchange reaction speeds up and becomes relevant at temperatures above *T*_v_, transforming the elastomer to a viscoelastic liquid of which the flow is mainly controlled by the cross-link exchange kinetics, giving the typical Arrhenian viscosity decrease. In the second example, an intrinsically fast exchange reaction is embedded in a rigid polymer matrix with a *T*_g_ that is higher than the expected *T*_v_ (Fig. 3b). In such cases, where *T*_v_ can be calculated *via* extrapolation of stress-relaxation or creep experiments, this transition is hypothetical since the network is not ultimately frozen by the reaction kinetics, but by the lack of segmental motions associated with *T*_g_. At temperatures below *T*_g_ no segmental motion occurs, consequently no exchange reactions can occur and the network is fixed (*cf.* diffusion limit). Upon heating above the glass transition region of the material, segmental motion is gradually initiated while the exchange reactions are already fast. In this initial situation, network rearrangement kinetics is diffusion-controlled and network topology rearrangements are dominated by segmental motions, which result in a WLF viscosity behavior. When heating further, the exchange kinetics change at a certain point from a diffusion controlled regime to an exchange reaction controlled regime, which follows the Arrhenius law.

For the design of vitrimer materials, it is important to consider the two transitions and their corresponding temperatures, *T*_g_ and *T*_v_, which can be controlled through parameters such as the cross-link density, intrinsic rigidity of monomers, the exchange reaction kinetics (*e.g.* catalyst loading), and the density of exchangeable bonds and groups. For most applications, vitrimers should behave as classical thermosetting polymer networks in a useful temperature window, *i.e.* without significant creep. Only when heated, the network reorganisation should become significant, resulting in a controlled macroscopic flow without risking structural damage. Thermal processing can even effectively repair defects, reminiscent to how classical materials such as metals or glass can be processed.

In the following two sections, the discussed polymer networks and their properties are subdivided into systems that either fully meet all vitrimer requirements and properties as discussed above (vitrimer materials) and systems that only show some of these properties (vitrimer-like materials).

## Vitrimer materials

In this section, an overview of the currently developed vitrimer materials is presented, with a subdivision based on the nature of the dynamic associative exchange reaction.

### Carboxylate transesterification

**Fig. 4:**
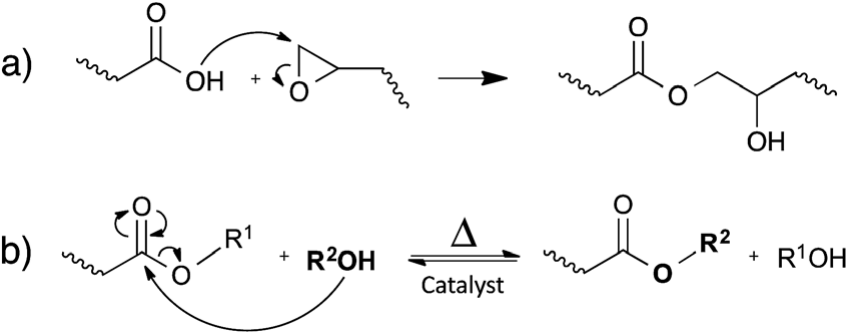
(a) The reaction between epoxides and carboxylic acids results in a repeating unit, containing both an ester and a hydroxyl function. (b) The catalytic transesterification reaction that can occur at elevated temperatures.

Leibler and co-workers initially demonstrated the concept of vitrimers by using simple carboxylic acid-based transesterification reactions, promoted by a catalyst (Fig. 4b). In classical epoxy/acid polymer networks, the abundance of both free hydroxyl functions and carboxylic esters is guaranteed by simply mixing stoichiometric amounts of bi- and poly-functional monomers (Fig. 4a). For epoxy/anhydride networks on the other hand, the polymerisation is more complex and the stoichiometry was carefully chosen, so that free hydroxyls are available throughout the network.^12^

Interestingly, the rate of the catalysed transesterification reaction at room temperature is insignificant. Hence, typical soft epoxy/acid networks (*T*_g_ below room temperature) perform like conventional elastomers at room temperature, without creep. However, upon heating, rapid exchange reactions allow the rearrangement of the network structure, enabling the material to be deformed, processed and recycled^19^ (*cf.* Fig. 3a).

One of the features of the transesterification-based vitrimers is the relatively straightforward control of the exchange reaction kinetics through catalysis. By changing the amount and nature of catalyst, the activation energy and *T*_v_ can be tuned with minimal perturbation of material properties. For example, activation energies for 1,5,7-triazabicyclo[4.4.0]dec-5-ene (TBD), zinc(ii)acetate (Zn(OAc)_2_), and triphenylphosphine (PPh_3_) are 106, 86 and 43 kJ mol^–1^ respectively.

In more recent work, Ji *et al.* demonstrated that also light can be used to process epoxy/acid-based transesterification vitrimers by dispersing 1 w/w% of carbon nanotubes (CNTs) into the polymer matrix.^20^ Carbon nanotubes absorb light of almost all wavelengths and transform the energy to heat, resulting in fast and precise local heating. CNT-impregnated vitrimers were successfully reshaped, healed and even welded with non-CNT-vitrimers *via* irradiation with an infrared laser.

As an alternative to epoxy-based transesterification vitrimers, Hillmyer *et al.* reported transesterification vitrimers prepared with isocyanate chemistry,^21^ using hydroxyl-terminated four-arm shaped polylactide and methylene diphenyl diisocyanate (MDI) monomers. A dioctyltin catalyst (Sn(oct)_2_) was used for both the polymerisation and the transesterification reactions. Compared to epoxy vitrimers, remarkably short relaxation times (50 s at 140 °C) were observed at all isocyanate/alcohol ratios, even when a small amount of free hydroxyl groups is present in the network. These short relaxation times can be attributed to the high concentration of ester groups in the network (polylactide backbone) and possibly to a higher catalyst activity. This result indicates that besides the amount of free hydroxyl groups, also the relative concentration or abundance of ester functions throughout the network matrix can influence the relaxation times tremendously.

Although all esterification-based vitrimers show good processability, long-term stability of the materials can be an issue through catalyst ageing or leaching, and ester hydrolysis. Especially the hydrophilic polylactide/urethane networks could have limited applications, as urethanes are known to absorb water.^22^

In summary, transesterification vitrimers, and especially epoxy-based ones, excel in availability of monomers and ease of synthesis, which makes them readily upscalable and applicable in an industrial context. On the other hand, fast processing is only achieved with high catalyst loadings and high temperatures. Moreover, catalyst (in)solubility becomes an issue, especially when rigid monomers are used for high *T*_g_ materials (>75 °C).^13^

In addition to the experimental work on transesterification vitrimers, theoretical models were also developed for these pioneering systems, leading to further insights in the remarkable dynamic behavior of these materials.^23,24^

### Transamination of vinylogous urethanes

**Fig. 5:**
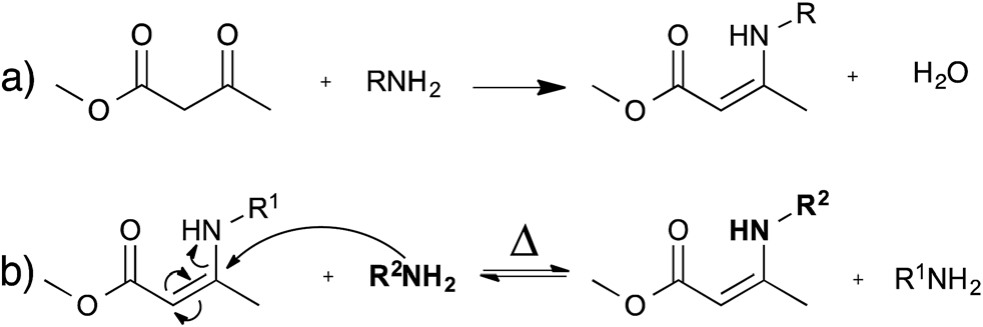
(a) Synthesis of vinylogous urethane; this reaction can even be performed in water. (b) Exchange of vinylogous urethanes and amines occurs at temperatures above 100 °C without catalyst *via* a Michael addition.

As an alternative to transesterification vitrimers, we recently explored vinylogous urethanes as a catalyst-free exchangeable group for vitrimers.^17^ From a thermodynamic point of view, the exchangeable carbon–nitrogen bond group is closely related to an amide or a urethane bond, because of the favorable electronic conjugation with the carbonyl function, mediated by the carbon–carbon double bond (*cf.* vinylogy principle).^25,26^ Vinylogous urethanes are thus stable towards hydrolysis, and can even be formed quantitatively in water as a solvent (Fig. 5a).^27^ From a kinetic point of view, the vinylogous urethane moiety also possesses the reactivity of a typical Michael acceptor through a facile conjugated nucleophilic addition of an amine group, displacing the less stable carbon–carbon double bond, rather than the much stronger carbonyl double bond. Indeed, swift exchange reactions at temperatures above 100 °C were demonstrated on low molecular weight compounds without catalyst (Fig. 5b).

Vinylogous urethane polymer networks have been prepared through a bulk polymerisation using the spontaneous condensation reaction between acetoacetate and amine monomers (Fig. 5a), with the release of water. Using slightly off-stoichiometric conditions, sufficient free amines are present in the polycondensation networks to allow swift exchange reactions. Indeed, the resulting materials showed good mechanical properties, similar to those of epoxy/acid vitrimers but with much shorter relaxation times (∼3 min at 150 °C). These rapid exchange dynamics can be attributed to the high density of vinylogous urethanes in the network, and to the low uncatalysed activation energy of ∼60 kJ mol^–1^. Despite these interesting features, the stability of the small amount of amines throughout the network needs to be taken into account for long-term applications, in view of possible oxidative damage.

### Transalkylation of triazolium salts

**Fig. 6:**
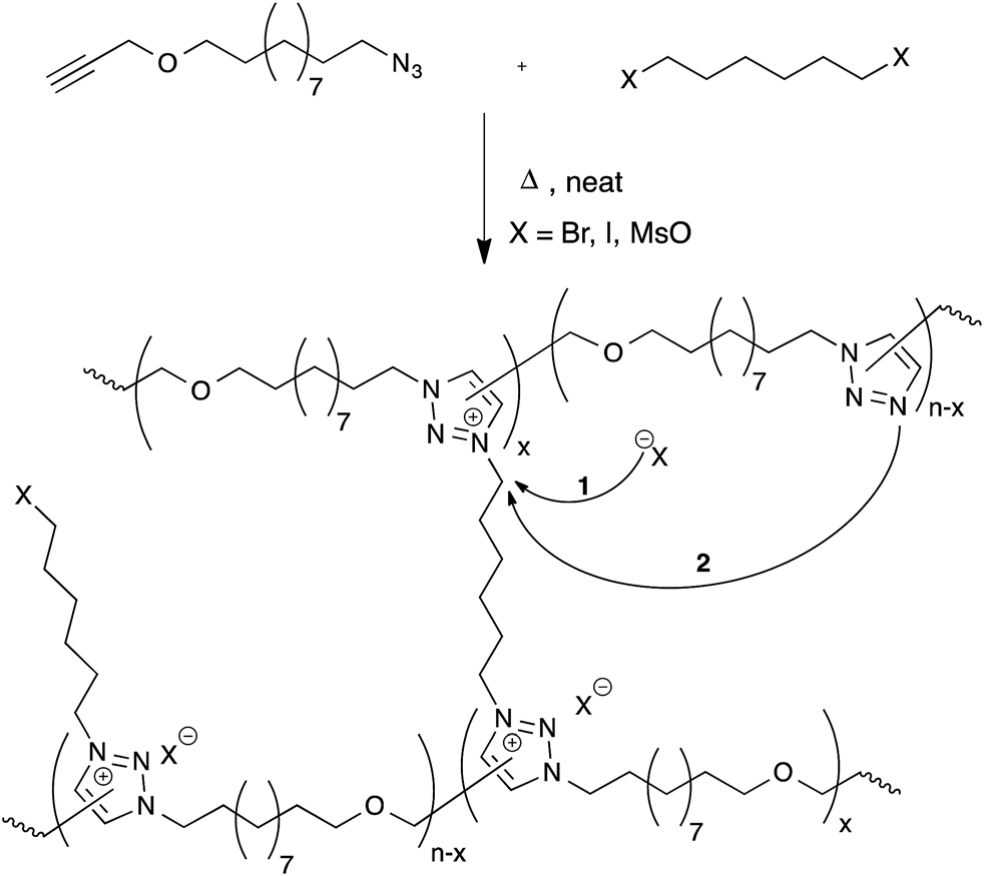
Polymerisation of α-azide-ω-alkyne monomers with bisfunctional alkylating agents results in polymer networks *via* the formation of 1,2,3-triazoles followed by the cross-linking process through alkylation to 1,2,3-triazoliums. The resulting networks can rearrange their topology through exchange between 1,2,3-triazoles and 1,2,3-triazoliums.

Drockenmuller and co-workers very recently reported new vitrimers based on transalkylation reactions in networks containing 1,2,3-triazolium salts, triazolines and pendant alkyl halide chains.^28^ Such polyionic networks were synthesized *via* a one-pot process through a polyaddition of α-azide-ω-alkyne monomers involving a thermal ‘non-click’ azide-alkyne Huisgen 1,3-dipolar cycloaddition and a simultaneous cross-linking step, using a bifunctional alkylating agent such as dibromo and diiodo alkanes or alkyl mesylates (Fig. 6). The resulting networks showed typical Arrhenian stress-relaxation with an experimental activation energy of 140 kJ mol^–1^ for the species with a bromide counter-ion. Short relaxation times ranging from 30 minutes at 130 °C to a few seconds at 200 °C were observed. Interestingly, flow properties in these systems can be controlled *via* the choice of counter-ion, as these can result in faster stress-relaxation (Br^–^ ≫ I^–^ > MsO^–^). The origin of the stress-relaxation through transalkylation reactions in the network was confirmed *via* model compound studies. Mechanistically, the exchange reaction is still unclear at this time, although two realistic pathways can be imagined. In one scenario, suggested by the authors, a group transfer can be mediated by the nucleophilic attack of a counterion (halide or mesylate) on an alkyl triazolium species (Fig. 6, arrow 1), which can then react with a different alkyl halide chain. This is a dissociative mechanism that should result in depolymerisation. However, this is not observed but might also be explained by substitution reactions that only happen within ‘pockets’ of ionic liquid-like ion pairs, preventing free diffusion of the new alkyl halide. In the other scenario, substitutions could also directly occur between alkyl triazolium salts and nucleophilic unalkylated triazoles, expelling a triazole and making a new triazolium species in a concerted S_N_2-type substitution (Fig. 6, arrow 2). As iodides are more nucleophilic than bromides, but triazolium bromides give faster exchange reactions, this associative exchange mechanism seems more likely and also more readily explains the observed vitrimer properties. However, more work will need to be performed on these intriguing systems in order to clarify the exact chemical mechanisms.

Despite the straightforward polymerization of triazolium salt polyionic networks, which is an easy, solvent- and catalyst-free one-pot process, the transalkylation vitrimers compare unfavorably to the former two systems in terms of scalability and cost, requiring hazardous and more expensive chemicals (azides and alkylating reagents). On the other hand, these polyionic materials offer an interesting functionality by their conducting properties, which can be quite useful for certain niche applications.

## Vitrimer-like materials

### Carboxylate transesterification

Williams and co-workers prepared networks from epoxidized soybean oil and citric acid in the presence of water, which are thus similar to Leibler's original system, using only renewable feedstock chemicals. Remarkably, these materials relax stresses and are healable, even without the addition of any catalyst.^29^ Since no catalyst is present, relaxation times are considerably higher, 5.5 h *versus* 2.4 h for epoxy/acid resins with 5% Zn(OAc)_2_ at 150 °C.^14^ However, full stress relaxation could not be achieved in these epoxy/acid resins, probably due to the formation of irreversible cross-links upon prolonged heating. The high abundance of free carboxylic acids, which is probably responsible for the catalysis of the transesterification reaction, also gives hydrophilic materials.

### Siloxane silanol exchange reaction

**Fig. 7:**
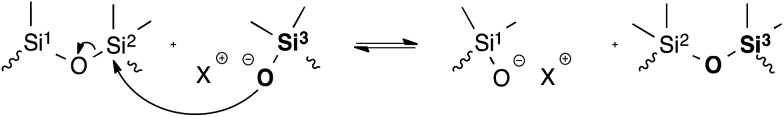
Siloxane silanol exchange reaction through addition and elimination of a silanolate anion.

In 2012, McCarthy *et al.* drew attention to the addition/elimination of silanols or silanolate on siloxane moieties as a ‘forgotten’ dynamic covalent bond forming reaction (Fig. 7), and demonstrated quantitative healing and stress-relaxation in polydimethylsiloxane (PDMS) networks.^30^ In fact, these results were already suggested in the 1950s^31–33^ and are also patented.^34^ In this report, qualitative stress-relaxation experiments showed that PDMS elastomers undergo siloxane rearrangements, catalysed by the presence of either acids or bases. While the exchange kinetics can be enhanced *via* these catalysts, degradation through hydrolysis and thermal cyclisation-depolymerisation becomes an issue.^35^ An interesting approach to avoid this degradation is the thermal decatalysation using a silanolate tetramethylammonium salt.^31^ The silanolate anion induces rapid exchange reactions even below 130 °C, but can be decomposed *via* thermal treatment at 150 °C, thus transforming the living polymer to a permanent elastomer. Although the vitrimeric properties of such ‘living’ polysiloxane networks remain to be explored, and thermal degradation seems to be an important issue in known systems, the associative nature of the covalent bond exchange reaction should allow for the design of new siloxane-based vitrimer materials.

### Olefin metathesis exchange reaction

**Fig. 8:**
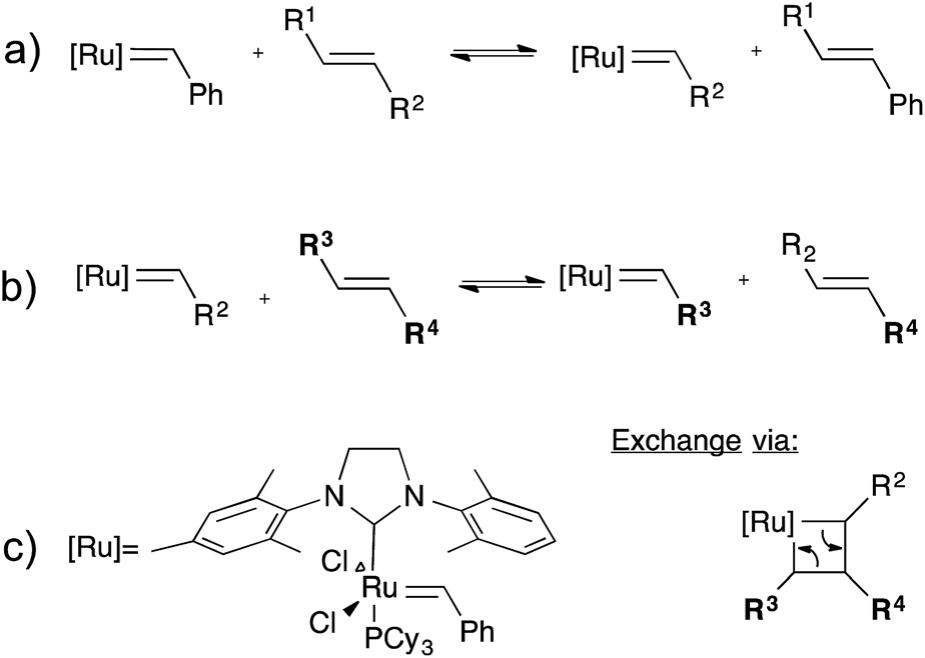
Olefin metathesis exchange reaction, (a) insertion of the Grubbs' second generation catalyst into an alkyl chain (b) exchange of two alkene bonds (c) left: structure of 2^nd^ generation Grubbs' catalyst; right: the associative exchange mechanism of a metathesis reaction.

While the olefin metathesis reaction is a very powerful tool for the formation of C–C bonds,^36–39^ and has found wide use in ring opening metathesis polymerisation (ROMP), cross-metathesis is much less used in polymer synthesis, as it lacks an internal driving force. For topology rearrangements *via* group exchange equilibria in networks, however, cross-metathesis should be a good method as no driving force is required. Indeed, Guan and co-workers demonstrated the possibilities of the olefin metathesis reaction in cross-linked polybutadiene networks containing second-generation Grubbs catalyst (Fig. 8).^40,41^ This catalyst was used because of its superior stability towards air and moisture and its good functional group compatibility, as compared to more classical metathesis catalysts.^42^ Networks were first prepared through free radical cross-linking of polybutadiene initiated by benzoyl peroxide. As the 2^nd^ generation Grubbs catalyst is not compatible with these cross-linking conditions, the catalyst needed to be introduced *via* a swelling experiment, which covalently incorporates the ruthenium catalyst by breaking some chains, also incorporating the original styrene-derived carbene ligand as a new chain end (*cf.* Fig. 8a). The obtained elastomeric ruthenium-bonded networks already showed significant stress-relaxation and creep at ambient temperatures, due to the highly effective exchange reaction. As expected, flow properties could be controlled *via* the catalyst concentration. For possible self-healing materials applications, this ambient exchange reaction is interesting since quantitative healing can be obtained, even at room temperature. For applications in typical vitrimer processing of rigid networks, the creep is highly undesirable for most applications where elastomers are typically used. Freezing the topology *via* the glass transition could be a solution to avoid creep, but may not be straightforward to implement. Furthermore, the sensitivity of the catalyst can also be used to ‘deactivate’ the catalyst after processing resulting in a permanent thermosetting elastomer.

### Disulfide exchange chemistry

The dynamic nature of sulfur–sulfur linkages and of disulfides^43–56^ in particular has been extensively studied because of the great interest for vulcanized rubbers in the chemical industry.^52,53^ Recent reports show that the behavior of covalently exchanging disulfide bonds is a rather complex process, and involves several mechanisms that also depend on the used conditions and substitution patterns of the disulfides. In the most simple form, disulfides can be reduced to two thiols and then oxidized again *via* a clearly dissociative stepwise pathway.^47–49^ Alternatively, disulfides can be homolytically but reversibly opened to stabilised thiyl radicals under action of UV,^56^ shear and/or heat,^50–52^ another dissociative but more direct pathway. An associative exchange is, in principle, possible by an addition/elimination substitution with free thiols.^46,54,57^

Recently, Goossens and Klumperman *et al.* ^54^ reported dynamic networks based on base-catalysed thiol-disulfide exchange reactions and could correlate the mechanical relaxation times with those of the model compounds, showing properties reminiscent of vitrimers, probably related to the associative exchange mechanism. However, as oxidation of free thiols readily occurs under air and free thiols are required for the exchange reaction, these materials show a marked deterioration of dynamic properties over time. The same exchange reaction has also been used by Klumperman,^46^ and by Zhang^57^ in previous studies on self-healing materials.

**Fig. 9:**
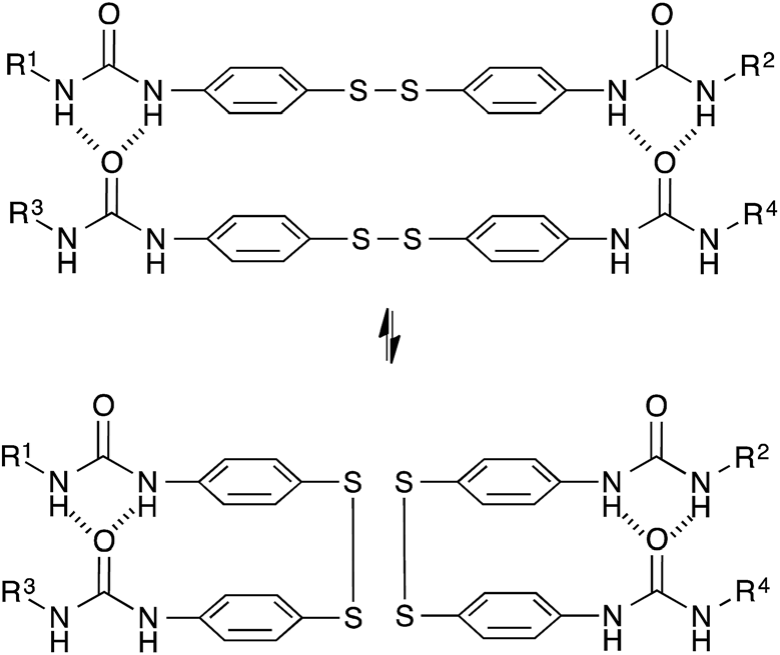
Aromatic disulfide metathesis proceeds at room temperature without any catalyst. The quadruple H-bonds of the urea groups prevent the network to flow at low temperatures.

Based on a different exchange mechanism, Odriozola *et al.* demonstrated processable elastomers using aromatic disulfide metathesis^43,44^ in poly(urea-urethane) networks. These networks showed quantitative self-healing at room temperature due to both dynamic hydrogen bond formation and network reshuffling (Fig. 9). Interestingly, while disulfide exchange reactions are known to occur rapidly at room temperature, little stress-relaxation is actually observed for deformations within the linear range of these materials at room temperature, as hydrogen bonds prevent the network to flow at low temperatures. The viscoelastic properties of these networks are thus more complex compared to vitrimers relying on a single relaxation process, since these two relaxation effects are superposed on each other.

### Imine amine exchange chemistry

**Fig. 10:**
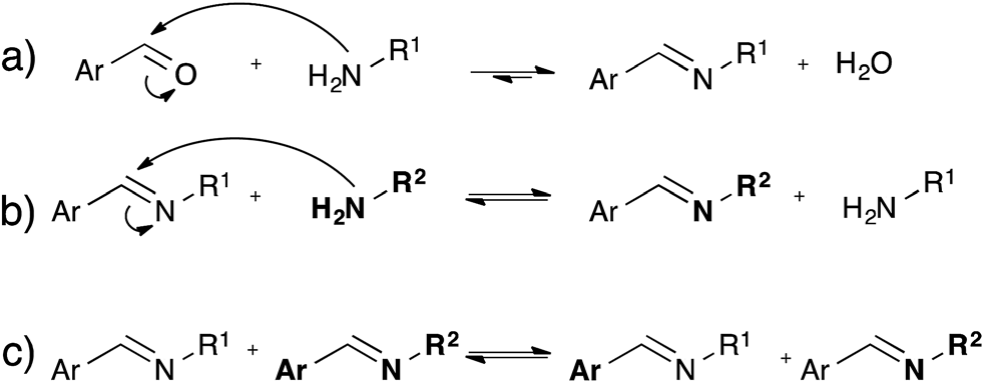
Reversible processes associated with imine chemistry. (a) Equilibrium of imine formation, *via* intermediate hemi-aminal formation; (b) transamination between imines and amines *via* intermediate aminal formation; (c) imine metathesis.

While the dynamic nature of imines in polymer chemistry is mainly exploited using its dissociative pathway involving reversible formation/hydrolysis of imines^58–60^ (Fig. 10a), also associative pathways in absence of water *via* either amine exchange^61^ (Fig. 10b) or imine metathesis (Fig. 10c) could result in a dynamic behaviour.^62^ Recently, Zhang and co-workers exploited associative imine chemistry for the design of malleable and recyclable polymer networks.^63^ These cross-linked polyimine networks were prepared *via* the condensation of commercially available aldehydes and a mixture of di- and triamines in a solvent combination that further drives the reversible imine forming condensation, which only has a very small intrinsic thermodynamic driving force. The stress-relaxation behavior of the dried polyimine networks exhibited Arrhenius-like temperature dependence together with water-induced stress-relaxation at room temperature, indicating vitrimer-like properties. While this malleability was assigned by the authors to a direct imine metathesis reaction (Fig. 10c), the fast stress-relaxation (30 min for 90% relaxation at 80 °C) can be more readily explained by a fast imine addition–elimination exchange through intermediate aminal formation with abundant free amines (Fig. 10b). Indeed, Di Stefano and co-workers have shown that even a minute amount of primary amines, which are very likely to be present in the polyimine network, induce very fast addition–elimination exchange reaction with imines.^61^ In addition, this swift transamination can also explain the fast water-induced stress-relaxation as the dissociative hydrolysis, (Fig. 10a) acting together with transamination induced by free amines, allows for very efficient network rearrangements. Despite the interesting possibilities of the room temperature water-induced malleability of these networks, the mechanical properties and useful applications of these polyimines networks are limited by this unavoidable sensitivity towards hydrolysis. Furthermore, in very recent work Zhang *et al.* reported a free radical metathesis-like exchange reaction of aromatic imine bonds for remoldable cross-linked polymers.^64^

## Conclusions and outlook

Classically, polymers are subdivided in two main classes according to their thermal behaviour: thermosets and thermoplastics. Since the introduction of vitrimers, a third class can be added. Ideally, these new materials behave as classical, insoluble static polymer networks at ambient temperatures. Upon heating, associative exchange reactions allow the materials to flow. Since the flow is controlled through chemical exchange reactions, the viscosity decrease is Arhennian, a feature reminiscent of vitreous silica that makes vitrimers the first reported strong organic glass formers. In contrast to dissociative CANs, vitrimers do not show thermoplastic behaviour at higher temperatures, and can thus not be simply regarded as a hybrid class that shows either thermoplastic or thermosetting properties depending on the temperature.

The unique viscoelastic properties of vitrimers originate from the thermally triggered associative exchange reaction, which installs permanent but dynamic cross-links throughout the bulk of the material. Consequently, in addition to the classical glass-to-rubber transition (*T*_g_), vitrimers possess a second transition, the topology freezing transition temperature (*T*_v_) that implies a change from a viscoelastic solid to a viscoelastic liquid due to the increasing rate of the exchange reactions. As the value of *T*_v_ indicates when vitrimers become processable but on the other hand also lose their resistance to creep, finding exchange reactions with the right kinetics toward the application is one of the main challenges for vitrimer research.

In this mini-review we show that several classical associative exchange reactions have already been used to design and produce vitrimers with unique properties. In addition, we show a range of other chemical systems that clearly show potential in this regard, if industrially relevant materials can be made based on them. This short but – at this time (June 2015) – comprehensive overview also shows that there are still plenty of opportunities to expand this exciting new class of organic polymer materials, which may lead to novel materials with unprecedented applications.

Ideal exchange reactions for vitrimers have predictable or controllable kinetics, in a wide temperature window, are stable towards the harsh conditions often used during processing, do not degrade over time, are upscalable and easily introduced in polymeric networks. Clearly, research into applications of different exchange reactions in vitrimer materials deserves attention from chemists with a wide range of backgrounds. From a synthetic point of view, dynamic associative equilibria have been underexplored in application-driven research, also pointing towards an area in need of development for fundamental methodological synthetic research. Furthermore, the concept of vitrimers should not be limited to covalent bonds but can also be expanded to supramolecular bonds.^65^

Apart from many unique applications such as reprocessable composites and liquid-crystalline elastomer actuators,^66^ vitrimers also hold the promise of changing the classical perception of bulk synthetic ‘plastics’. Today, society generally regards organic polymers as cheap, fit-for-purpose discardable materials that ultimately become a hazardous ‘chemical’ waste. As vitrimers can in principle be easily (re)processed, recycled and repaired in a similar way to glass and metals, bulk synthetic organic polymers might also evolve towards a new perception as versatile light weight raw materials with a high intrinsic value. For this, chemists need to expand and develop the polymer chemistry toolbox with innovative ways to reliably and easily control the intrinsic chemical reactivity of bulk organic materials.

## Note added in proof

In very recent work by Guan *et al.*, the transesterification of boronic esters has been studied as dynamic bonds for malleable materials.^67^
